# Mechanically powered negative pressure dressing reduces surgical site infection after stoma reversal

**DOI:** 10.1016/j.sopen.2025.01.002

**Published:** 2025-01-15

**Authors:** Brian Williams, Aubrey Swinford, Jordan Martucci, Johnny Wang, Jordan R. Wlodarczyk, Abhinav Gupta, Kyle G. Cologne, Sarah E. Koller, Christine Hsieh, Marjun P. Duldulao, Joongho Shin

**Affiliations:** a Keck Medicine of USC, Division of Colorectal Surgery, Los Angeles, CA, USA; b Keck School of Medicine of USC, Los Angeles, CA, USA; c Los Angeles General Medical Center, Division of Colorectal Surgery, Los Angeles, CA, USA

**Keywords:** Negative pressure dressing, Closed-incision negative pressure wound therapy, Surgical site infection, Wound healing, Colorectal, Ileostomy closure, Colostomy closure

## Abstract

**Background:**

The use of closed-incision negative pressure wound therapy (ci-NPWT) has been shown to reduce postoperative wound complications and surgical site infections (SSI) after stoma closures. However, use of this approach has not been widely adopted due to high cost of the devices. We present a novel approach to stoma closure in which a self-contained mechanically powered negative pressure dressing (MP-NPD) is applied to primarily closed stoma reversal wounds. We hypothesized that SSI and wound complication rates would be improved compared to traditional stoma closure methods.

**Methods:**

This was a prospective investigator-initiated study, in which consecutive patients that underwent stoma reversal with primary stoma wound closure dressed with MP-NPD from May 2021–March 2022. 30-day outcomes from the study group, including surgical site infection, other wound complications, hospital length of stay (LOS), and readmission rates, were then reported.

**Results:**

Forty-six patients undergoing local ileostomy or colostomy closure were identified for the study group. Patient demographics and surgical variables were reported. One (2.2 %) patient in the study cohort developed superficial SSI within 30 days of their surgery. Post-op LOS in the study group versus was 4.1 days.

**Conclusion:**

Intestinal stoma reversal wounds closed primarily and dressed with the MP-NPD dressings had very low stoma site SSI rates. These results are promising as they pertain to the use of MP-NPD in stoma reversal procedures, however further large prospective RCTs with a matched control group could help better corroborate these findings.

## Highlights

• MP-NPD reduces SSI risk in stoma reversal wounds.
• Significant decrease in wound dehiscence with MP-NPD use
• Study shows shorter hospital stay with MP-NPD.
• MP-NPD improves post-surgical wound healing outcomes.

## Introduction

Intestinal stoma creation is a commonly performed procedure with a variety of surgical indications. These stomas are often intended to be temporary, allowing proper time for anastomotic healing and patient recovery. As such, stoma reversal is also a frequently performed operation. Perioperative complication rates after stoma reversal can be as high as 13 % for ileostomies and even greater for colostomy reversal [1,2]. Surgical Site infection (SSI) is a risk inherent in the operation, with SSI rates reported as high as 40 % in conventionally closed stoma reversal wounds [3]. As such, various techniques, antibiotic prophylaxis regimens, and wound therapies have been used to minimize the risk of SSI in these patients. One of the most studied and accepted skin closure techniques used to address risk of SSI is the purse-string closure, which has SSI rates as low as 5 % [4]. While patient satisfaction is acceptable with this technique, inherent in this type of closure is the need for frequent dressing changes and diligent wound management during the healing process, which may be unacceptable or difficult for some patients. Antibiotic impregnated sutures have also been developed to decrease SSI risk; however, their use has not shown significant benefits in a variety of abdominal surgical procedures and are not frequently used for stoma reversals [5].

In recent years, negative pressure wound therapy (NPWT), delivered through a wound vacuum-assisted closure (VAC) device, has been used successfully to treat complex open wounds. More recently NPWT has been used prophylactically on primarily closed wounds and has demonstrated decreased SSI, wound complications, and shorter healing time in a variety of specialties [[6], [7], [8]]. Initial studies assessing the utility of closed incision NPWT (ci-NPWT) in colorectal surgery procedures have also shown reduction in the incidence of SSI for laparotomy and perineal wounds after open colectomy and abdominoperineal resection, respectively [9,10]. Subsequent studies assessing the role of ci-NPWT for stoma reversal wounds have also suggested lower SSI rate and wound healing time [[11], [12], [13], [14]].

Despite increased use of NPWT, the wide adoption of prophylactic ci-NPWT has been slow due to higher costs and complexity of use associated with standard systems [[15], [16], [17]]. The NPseal© (Guard Medical, Miami, FL, USA) is a self-contained, mechanically powered negative pressure dressing (MP-NPD) that can achieve therapeutic negative pressure when applied over primarily closed surgical wounds. These MP-NPDs provide ci-NPWT comparable to the standard systems with potentially wider applications due to limited cost and ease of use. Pricing of the device varies based on individual contract negotiations with hospitals, but typically range from one-fourth to one-third the cost of other powered negative pressure devices. Our colorectal surgery division has used this device successfully on a wide variety of surgical incisions. However, its prophylactic use for primarily closed stoma reversal wounds had not been specifically evaluated. The purpose of this study was to assess 30-day wound complication and stoma site SSI rates of patients undergoing primary closure of stoma reversal wounds followed by placement of this MP-NPD. We hypothesized that this novel usage of MP-NPD in stoma reversal wounds would improve SSI and wound complication rates when compared to traditional stoma closure techniques.

## Materials and methods

### Study design

This was a subgroup analysis of patients enrolled as part of a larger prospective, single center, observational study, assessing outcomes of primarily closed wounds dressed with a novel MP-NPD.

### The mechanically powered negative pressure dressing (MP-NPD)

**Fig. 1:**
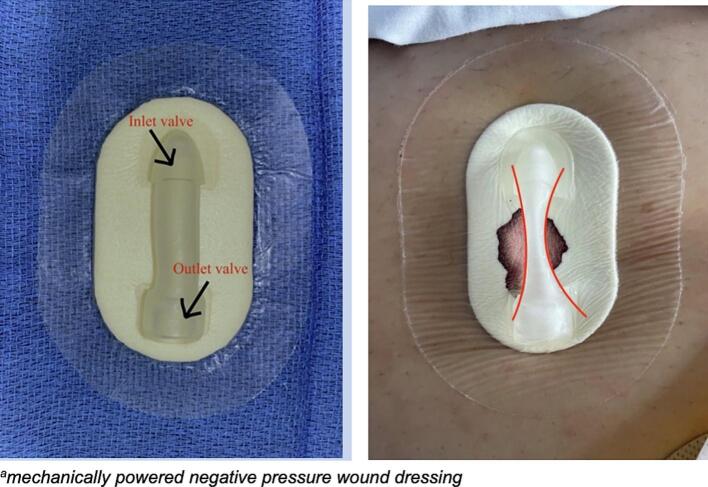
Inactivated (left) and activated (right) MP-NPD^a^ dressings. ^a^Mechanically powered negative pressure wound dressing.

The MP-NPD used in the study group was the NPSeal® (Guard Medical, Miami, FL, USA), which is an FDA approved self-contained MP-NPD. It features an integrated pump consisting of an inlet valve, which communicates with the dressing pad, and an outlet valve through which air exits. After placement of the dressing, the pump body is pinched until it remains collapsed, resembling an “hourglass” shape, indicating therapeutic pressure between −75 and − 125 mmHg (Fig. 1). The dressing can be left in place for up to 6 days. While in place, the pump is monitored by clinical staff and/or patients to confirm it remains collapsed. If it is de-compressed, the pump can be pinched until it collapses again.

### Study cohort

Patients included in the study group were those that underwent elective colostomy or ileostomy reversal between May 2021 and March 2022 at our institution. Each patient had the MP-NPD applied to a primarily closed ileostomy or colostomy reversal wound. The dressings were left in place for up to 6 days. If >50 % saturation of dressing, or unable to maintain adequate adhesive seal, the MP-NPD could be replaced up until day 6. The wounds were evaluated by a clinician at time of dressing removal, and all 30-day outcomes were reported by the patient and/or observed by the surgical team during routine postoperative evaluations and documented in the health record. Clinicians assessing wounds for SSI were not blinded to the closure technique during the initial wound assessment at 6–7 days or at 30-days and clinic visits.

### Compliance with ethical standards

The study was approved by the Institutional Review Board at the University of Southern California, under IRB ID HS-16-00812. All prospectively enrolled participants in the study group provided written informed consent before partaking.

### Primary outcomes

The primary outcome of this study was 30-day stoma SSI rate after closure. SSI was defined based on the Center for disease Control and Prevention (CDC) definition of a superficial or deep SSI at or near the incision within 30 days of the operation. Clinically, an infection was defined as presence of incisional purulent drainage, erythema, warmth, pain, or wound breakdown not otherwise explained by other causes. All patients were provided 24-h access to a department hotline and encouraged to contact staff if there were any suspected surgical issues during recovery.

### Secondary outcomes

Wound outcomes including skin dehiscence, seroma/hematoma formation, blistering, and skin excoriation were assessed. Other relevant patient, perioperative, and 30-day clinical outcomes data were collected via chart review of the electronic health record and operative reports for the patients in the study group.

### Perioperative preparation and operative technique

Patients underwent standard preoperative preparations following recommended surgical care improvement project (SCIP) guidelines. In the operating room, the skin was prepped with a combination of chlorhexidine and/or iodine skin prep. Patients received antibiotic prophylaxis within an hour of incision, usually with 1 g of intravenous Ertapenem or an equivalent regimen for patients with known allergies to carbapenems. Ertapenem was chosen as the typical antibiotic as this is the standard antibiotic used for elective colorectal surgery cases at our institution followed by levofloxacin plus metronidazole if there were contraindications to carbapenems. Other antibiotics were used if indicated as part of a combined procedure. The fascia was closed with 0 polydioxanone (PDS™ [Ethicon Inc., Somerville, NJ, USA]) suture in a running or interrupted fashion. The subcutaneous tissue was closed using interrupted polyglactin 910 (Vicryl™ [Ethicon Inc., Somerville, NJ, USA]) suture and the superficial skin was closed with running subcuticular 4–0 poliglecaprone 25 (Monocryl™ [Ethicon Inc., Somerville, NJ, USA]) suture. All wounds were irrigated with warm saline, iodine solution, sterile water, or a combination prior to skin closure. This was then followed by placement of the MP-NPD dressing, and the pump was squeezed until satisfactory negative pressure was maintained. The therapeutic window for the dressing was up to 6 days and was removed by the clinical team if the patient was in the hospital at postoperative day 6. If the patient was discharged prior to removal, they were instructed to remove the dressing on postoperative day 6 or sooner if needed.

### Statistical analysis/considerations

All statistical analyses were performed using the IBM SPSS Statistics software ver. 28.0 (SPSS Inc., Chicago IL, USA). Continuous variables were described using mean and standard deviation (st dev) or median and interquartile range (IQR), depending on normality. Categorical variables were described using frequencies and percentages. We used a two-tailed *p*-value of <0.05 to define statistical significance.

## Results

Forty-six consecutive patients undergoing local ileostomy or colostomy closures were enrolled and included in the analysis. The average age was 55.6 ± 13.9 years, and 23 (50 %) patients were female. The average body mass index (BMI) was 27.3 ± 7.7 and the median ASA grade was 3 (range 1–3). Nearly all patients were ASA grade 2 or 3 (45.7 % and 52.2 %, respectively). Four (8.7 %) patients reported current tobacco use in the perioperative setting, 9 (19.6 %) reported a history of tobacco use, and 3 (6.5 %) had diabetes. 23 patients (50.0 %) received systemic chemotherapy for rectal or colon cancer. The average time between cessation of chemotherapy and stoma closure was 218.5 days. Forty (87.0 %) patients underwent ileostomy reversal and 6 (13.0 %) underwent colostomy reversal. Two patients (4.3 %) required conversion to open laparotomy due to extensive adhesions and inability to fully mobilize the bowel through a local incision. Only the stoma wounds were evaluated, even in those patients' requiring conversion to an open procedure. The majority of patients (56.5 %) required temporary diverting stoma as part of a colorectal cancer procedure, followed by diversion for management of leak, infection, or fistula (26.1 %), and inflammatory bowel disease (13.0 %). One (2.2 %) patient required diversion due to an iatrogenic injury and 1 (2.2 %) required diversion due to severe hidradenitis. Details of demographics and preoperative characteristics of the study cohort are shown in Table 1.

**Table 1:** Patient demographics/preoperative characteristics.

Preoperative Characteristic	Total (***N*** = 46)
Age, years [avg (sd)]	55.6 (13.9)
BMI, kg/m^2^ [avg (sd)]	27.3 (7.7)
Systemic Chemotherapy, [n (%)]	23 (50)
Female, [n (%)]	23 (50)
Tobacco Use, [n (%)]	13 (28.3)
Diabetes, [n (%)]	3 (6.5)
ASA Class	
1	1 (2.2)
2	21 (45.7)
3	24 (52.2)
4	0 (0)
Stoma Type, [n (%)]	
Ileostomy	40 (87.0)
Colostomy	6 (13.0)
Indication/Reason for Stoma, [n (%)]	
Cancer	26 (56.5)
IBD	6 (13.0)
Leak/infection/Fistula	12 (26.1)
Hidradenitis	1 (2.2)
Iatrogenic Injury	1 (2.2)

avg, average; sd, standard deviation; BMI, body mass index; IBD, inflammatory bowel disease; ASA, American Society of Anesthesiologists.

### Perioperative/surgical data

Forty-three (93.5 %) patients received ertapenem or levofloxacin/metronidazole for their prophylactic antibiotics. Notably, 2 patients received piperacillin-tazobactam per hepatobiliary surgery preference during a combination procedure and 1 (2.2 %) patient received cefazolin during ileostomy reversal and ventral hernia repair combo procedure. Adequate antibiotic prophylaxis was provided in 42 (91.3 %) of cases. The 4 (8.7 %) patients that did not have adequate antibiosis were due to inadequate re-dosing of antibiotics during a prolonged combo surgery. Antibiosis for the colorectal portion (stoma reversal) was adequate in all cases on review of operative reports and operative room records. Skin preparation used was a combination of chlorhexidine and iodine skin prep in 38 (82.6 %) cases, with 4 (8.7 %) receiving iodine prep to skin and stoma, and 4 (8.7 %) receiving chlorhexidine to skin only. The average operative duration for the study cohort was 83.4 ± 55.4 min. The interval from stoma creation to reversal was 28.9 ± 31.6 weeks. Average intravenous fluids were 1025 ± 804 ml (ml), average estimated blood loss (EBL) was 81.3 ± 248.1 ml, and 1 (2.2 %) patient required intraoperative blood transfusion. All wounds were irrigated prior to skin closure, with most cases (60.9 %) irrigated with warm saline, followed by warm saline and iodine mixture (34.8 %). One (2.2 %) patient had their wound irrigated with iodine solution only and 1 (2.2 %) had their wound irrigated with sterile warm water. Sixteen patients had at least one other procedure performed during stoma reversal. Most cases with an additional procedure performed included flexible sigmoidoscopy with or without anal/anastomotic dilation. Further details regarding additional procedures performed and operative characteristics can be seen in Table 2.

**Table 2:** Surgical variables.

Intraoperative Variable	Total (N = 46)
Prophylactic Antibiotic Given, [n (%)]	
Ertapenem	38 (82.6)
Levaquin/Metronidazole	5 (10.9)
Piperacillin-Tazobactam	2 (4.3)
Cefazolin	1 (2.2)
Adequate Antibiotic Prophylaxis^a^, [n (%)]	42 (91.3)
Skin Preparation	
Iodine only	4 (8.7)
Chlorhexidine only	4 (8.7)
Chlorhexidine & Iodine	38 (82.6)
Operative Time, minutes [avg (sd)]	83.4 (54.8)
Intravenous Fluids, milliliters [avg (sd)]	1025 (804)
Blood Transfusion, [n (%)]	1 (2.2)
Wound Irrigation, [n (%)]	
Warm Saline	28 (60.9)
Iodine	1 (2.2)
Warm Saline & Iodine	16 (34.8)
Sterile Water	1 (2.2)
Other Procedure Performed, [n (%)]	16 (34.8)
Flexible Sigmoidoscopy	8 (50.0)
Anal/Anastomotic Dilation	6 (37.5)
Laparotomy/Lysis of Adhesions	2 (12.5)
Hernia Repair	2 (12.5)
Other Bowel Resection	2 (12.5)
Liver Resection	3 (18.8)
Port-a-Cath Removal	2 (12.5)
Interval to reversal, weeks [avg (sd)]	28.9 (31.6)

avg, average; sd, standard deviation.

a 4 patients without adequate antibiotics were not re-dosed at appropriate interval during prolonged surgery.

### Postoperative outcomes

The average hospital length of stay (LOS) was 4.1 ± 4.2 days. The 30-day readmission rate was 6.5 %. In regard to the primary wound outcome, there was 1 (2.2 %) patient that developed stoma site SSI within 30 days of the operation. There were 6 other minor wound complications in 5 (10.9 %) patients. Two (4.3 %) patients in the study group developed seroma and 2 (4.3 %) experienced superficial wound dehiscence. One patient in the study group had a small 1 cm superficial dehiscence due to seroma formation, which spontaneously drained without signs of underlying infection and was successfully managed with conservative measures. A second patient developed superficial skin dehiscence of the entire wound and associated superficial SSI which required removal of MP-NPD dressing prior to discharge and management with wet to dry gauze packing. This patient had complete wound healing by the time of first follow-up visit managed conservatively and did not require added antibiotic treatment. Additionally, there were 2 (4.3 %) patients who developed minor wound blistering at the adhesive edges of the MP-NPD dressings. These blisters were managed conservatively with local wound care and dry dressings as needed with resolution by time of first follow-up visit. All wound complications were classified as Clavien-Dindo Class I [18]. Further details of perioperative results can be seen in Table 3.

**Table 3:** 30-Day outcomes.

Outcome	Total (N = 46)
Postoperative LOS, days [avg (sd)]	4.1 (4.2)
Wound Complication^a^, [n (%)]	
Surgical Site Infection^b^	1 (2.2)
Seroma^c^	2 (4.3)
Superficial Skin Dehiscence	2 (4.3)
Skin Blistering	2 (4.3)
30-Day Readmission, [n (%)]	3 (6.5)

LOS, length of stay; sd, standard deviation; avg., average.

a All complications Clavien-Dindo Class I.

b Ileostomy reversal.

c Seroma fluid sterile or not sent for culture.

## Discussion

Intestinal stoma reversal procedures are often falsely thought of as “minor” procedures. This can be misleading as the degree of enterolysis and bowel manipulation can be extremely variable, with complication rates as high as 13.5 % for ileostomy reversal and 43.8 % for an end colostomy reversal [1,2]. Among the complications, SSI remains one of the most common after stoma reversal. As such, multiple strategies and surgical techniques have been employed in attempt to reduce this risk. Typical surgical techniques used for superficial wound closure include conventional primary wound closure, purse string closure (PSC), and delayed closure (DC). Unsurprisingly, the rate of SSI is significantly reduced when opting for a closure technique other than primary wound closure, such as PSC or DC [19,20]. In fact, a recent large metanalysis by Hajibandeh et al. [4] reviewed outcomes from 9 RCTs comparing outcomes of PSC versus primary linear wound closure of 757 patients undergoing stoma reversal procedures. This analysis showed that absolute 30-day SSI risk for PSC was 52 per 1000 people compared to 243 per 1000 people who underwent primary linear closure alone. On the other hand, some studies have shown that techniques like DC can lead to increased costs, longer time to healing, and diminished cosmetic results, which also need to be considered when performing any surgical procedure [20].

Another technique more recently introduced is the use of prophylactic NPWT over a primarily closed incision (ci-NPWT). Review of the literature shows encouraging results in a variety of specialties [[6], [7], [8], [9], [10]]. More recently, the use of ci-NPWT after intestinal stoma reversal has been studied and shown to have faster time to healing, decreased wound complication and SSI rates, and overall improvement in patient satisfaction compared to other standard closure techniques [11,21,22]. However, the ci-NPWT systems used in these studies relied on more traditional, bulky NPWT and VAC systems. Our current study is unique in that it is the first to assess wound healing outcomes of ci-NPWT using this novel MP-NPD. The benefit of this dressing is that it removes the need for a bulky VAC device to maintain negative pressure. To date there have been no other studies evaluating outcomes of patients undergoing stoma reversal with ci-NPWT using similar MP-NPD systems.

Our results show that for patients undergoing elective colostomy and ileostomy reversal, ci-NPWT with the MP-NPD had a very low 30-day stoma site SSI rate. In fact, only 1 (2.2 %) patient developed SSI within 30 days of reversal, which is in stark contrast to the 30-day SSI rates reported in the literature for conventional linear closure which range from 9 to 39 % [4]. Importantly, the low SSI rate in this current study is comparable to other well-established closure techniques like PSC, which is often preferred for its low SSI rate, which is around 5 % [4].

Additionally, the use of a MP-NPD was well tolerated by patients in the study cohort, with a low rate of minor wound complications, which mainly included skin irritation and blister formation. Review of intraoperative dressing placement technique also revealed that blister formation was at least partially due to overstretching of the dressing during application by the surgical team, which lead to unnecessary traction at the adhesive edges. Blister formation was not associated with the pump portion of the dressing. In general, blister formation with adhesive dressing placement has been shown to occur frequently in certain elective surgeries. Cosker et al. [23] reported on wound care outcomes for patients undergoing orthopedic procedures in a prospective study comparing different dressing types and found a blister formation rate of 16 % for a commonly used adhesive dressing and even higher rates (up to 24 %) for other types of dressings. This is substantially higher than the blister formation rate in our study of 4.3 %. Importantly, all blister formation resolved by the time of first clinic follow-up visit and all wound complications were managed conservatively with satisfactory results.

Finally, in the current study the standard perioperative antibiotic prophylaxis included ertapenem given intravenously within 1 h of incision. While this is an acceptable form of SSI prophylaxis, the use of ertapenem may be considered unusual as the standard for many surgeons that often utilize a combination of antibiotics, such as a combination of cephalosporin or fluoroquinolone plus metronidazole. The use of carbapenems have been shown to provide reduction of SSI in patients with fecal colonization by extended spectrum beta lactamase (ESBL) producing microbes when compared to patients treated with cephalosporin-based antibiotics [24]. This is becoming increasingly important as the prevalence of ESBL producing Enterobacteriaceae (ESBL-PE) colonization in the gut of healthy adults is rising. In fact, ESBL-PE colonization has risen as much as 5.4 % per year in some studies and may be as high as 46 % in some regions [25]. As such, the use of ertapenem prophylaxis may contribute to a lower SSI rate in the current study if prevalence ESBL-PE carriers is significant, but this was not evaluated in the current study. Indeed, there is data to support improved SSI prevention when using carbapenem antibiotic prophylaxis during colorectal procedures, when compared to other single agent prophylaxis regimens [26,27]. However, other studies comparing typical antibiotic prophylaxis regimens to carbapenems in colorectal surgery have not supported these findings [28,29]. Overall, the common use of ertapenem in this current study likely had little impact on the observed 30-day SSI rate.

### Limitations

We acknowledge that there are several major limitations inherent in this study which need to be taken into consideration when interpreting the findings. First, the sample size of our study is relatively small with <50 patients observed. However, despite this apparent small sample size, when analyzing available RCTs in the literature, the sample size per treatment arm ranges from 14 to 96 patients, with most studies including 40 patients or less per study arm [3,4,[30], [31], [32], [33], [34], [35], [36]]. As such, the sample size in our study is slightly larger than those of other prospectively collected samples available in the literature. Another limitation of our study is the study design as a single arm observational cohort study. While the data collection was performed in a prospective nature, the observational method without an appropriately matched control group decreases the ability to attribute any causality of our findings and the results of the study should be taken with caution. Additionally, the observational design limits the ability to perform true treatment randomization, which introduces the possibility of selection bias, which may also skew the observed findings of this study. Despite the limitations, this current study is the first of its kind to report on surgical site/wound outcomes using a novel MP-NPD as part of ci-NPWT closure method in stoma reversal surgery, and the results raise important questions regarding optimal wound closure methods for these procedures.

## Conclusion

Use of a MP-NPD dressing on primarily closed ileostomy and colostomy reversal wounds was feasible and safe with a very low stoma site SSI rate. The application of this simple negative pressure dressing should be considered for patients undergoing primary wound closure after intestinal stoma reversal. Further large prospective RCTs are needed to better evaluate this technique compared to more universally accepted stoma closure methods to better corroborate these findings. Current prospective RCTs are underway at our institution to further evaluate time to wound closure and complete wound healing when comparing conventional purse-string stoma closures to primary stoma closure and placement of MP-NPD.

## CRediT authorship contribution statement

**Brian Williams:** Writing – review & editing, Writing – original draft, Formal analysis. **Aubrey Swinford:** Writing – review & editing, Investigation, Formal analysis. **Jordan Martucci:** Writing – review & editing, Investigation, Data curation, Conceptualization. **Johnny Wang:** Writing – review & editing, Investigation, Data curation, Conceptualization. **Abhinav Gupta:** Writing – review & editing, Formal analysis. **Kyle G. Cologne:** Writing – review & editing, Supervision. **Sarah E. Koller:** Writing – review & editing, Supervision. **Christine Hsieh:** Writing – review & editing, Supervision. **Marjun P. Duldulao:** Writing – review & editing, Supervision. **Joongho Shin:** Writing – review & editing, Supervision.

## Ethical approval statement

The study was approved by the Institutional Review Board at the University of Southern California, under IRB ID HS-16-00812. All prospectively enrolled participants in the study group provided written informed consent before partaking.

## Funding sources statement

This research did not receive any specific grant from funding agencies in the public, commercial, or not-for-profit sectors.

## Declaration of competing interest

The authors declare that they have no known competing financial interests or personal relationships that could have appeared to influence the work reported in this paper.
